# Mechanisms of formation of emotional burnout syndrome in psychiatrists during the war in Ukraine

**DOI:** 10.1192/j.eurpsy.2026.11843

**Published:** 2026-07-31

**Authors:** N. O. Maruta, T. V. Panko, V. Y. Fedchenko, I. O. Yavdak, O. E. Semikina

**Affiliations:** Borderline Psychiatry, “Institute of Neurology, Psychiatry and Narcology named after P. V. Voloshyn of NAMS of Ukraine” SI, Kharkiv, Ukraine

## Abstract

**Introduction:**

Psychiatrists constitute a specific risk group for the development of Burnout. During wartime, this situation worsens due to increased workload, responsibility, and the inability to engage in meaningful recreational activities.

**Objectives:**

To determine the mechanisms of Burnout formation in psychiatrists during the war in Ukraine.

**Methods:**

MBI burnout scale by K. Maslach and S. Jackson, “Professional Life Quality Scale PROQOL-5”, and “Coping Strategies” questionnaire by R. Lazarus.

**Results:**

210 psychiatrists from various psychiatric institutions in Ukraine were surveyed. According to the results of the MBI burnout scale by K. Maslach and S. Jackson, signs of burnout of varying degrees of severity and at different stages of development were identified in 93.35% of psychiatrists, which creates risks of increasing the intensity of Burnout manifestations.

The assessment of the quality of professional life shows that for all three indicators (Compassion Satisfaction scale, Burnout scale, Compassion Fatigue/Secondary Traumatic Stress scale), average scores were obtained (32.94, 28.54, and 26.87 points, respectively), which indicates a negative impact of workload on well-being.

The data obtained using the method of R. Lazarus indicate that the coping strategies “Distancing” (7.03 points), “Self-control strategy” (7.37 points), “Search for social support” (8.04 points), “Planning to solve the problem” (8.27 points), “Positive reappraisal” (12.99 points), “Escape-avoidance” (10.98 points) have average indicators, which indicates the marginal level of the adaptive potential of the individual. Only the coping strategies “Accepting responsibility” and “Confrontational coping” have low indicators, which indicates the adaptive version of these coping strategies.

**Conclusions:**

The data obtained indicate that most psychiatrists during the war showed signs of Burnout. In the mechanisms of SEV formation, working conditions and personal coping strategies are of great importance; the vast majority of surveyed psychiatrists indicate a marginal state of adaptive potential and a risk of maladaptation. The changes identified indicate the need for psychocorrectional work to improve the psychological stability of psychiatrists during the war.

**Disclosure of Interest:**

None Declared

